# Supplementary material for: Identification of Metabolomics Biomarkers in Extracranial Carotid Artery Stenosis
Source: Cells. 2022 Sep 27;11(19):3022. doi: 10.3390/cells11193022 (PMC9563778; doi:10.3390/cells11193022)
Supplement: Supplementary file 1 [file cells-11-03022-s001.zip › 20220927 Supplemental Table S1- THL.pdf]

Identification of metabolomics biomarkers in extracranial carotid artery stenosis

Chia-Ni Lin [1,2], Kai-Cheng Hsu [3], Kuo-Lun Huang [4,5], Wen-Cheng Huang [6], Yi-Lun Hung [6] and Tsong-Hai Lee [4,5]

[1] Department of Laboratory Medicine, Linkou Chang Gung Memorial Hospital, Taoyuan 333, Taiwan

[2] Department of Medical Biotechnology and Laboratory Science, Chang Gung University, Taoyuan 333, Taiwan

[3] School of Medicine, College of Medicine, Artificial Intelligence Center for Medical Diagnosis, and Department of Neurology, China Medical University Hospital, Taichung 404327, Taiwan

[4] Stroke center and department of Neurology, Linkou Chang Gung Memorial Hospital, Taoyuan 333, Taiwan

[5] College of medicine, Chang Gung University, Taoyuan 333, Taiwan

[6] Department of Nuclear Medicine, Linkou Chang Gung Memorial Hospital, Taoyuan 333, Taiwan

Corresponding author:

Tsong-Hai Lee, MD, PhD

Professor of Neurology, Linkou Chang Gung Memorial Hospital, Taoyuan, Taiwan.

No. 5, Fu-Hsing street, Kweishan, Taoyuan, 333, Taiwan

Tel: +886-3-3281200, ext. 8340

e-mail: thlee@adm.cgmh.org.tw

Supplementary Table S1. Comparison of metabolite levels between normal controls and the 3 subgroups of carotid artery stenosis

Amino acid (μM) / No.	A. Plaque score = 0	B. Plaque score ≥ 2	Adj P_{FDR} A vs. B	C. Plaque score ≥ 5	Adj P_{FDR} A vs. C	D. Plaque score ≥ 8	Adj P_{FDR} A vs. D
	127	173		142		79	
**Acylcarnitines** (40)							
C0	37.531 ± 10.730	41.780 ± 15.584	0.017*	42.296 ± 16.251	0.016*	43.822 ± 17.843	0.024*
C10	0.222 ± 0.105	0.174 ± 0.096	5.588×10^{5}*	0.178 ± 0.099	0.003*	0.189 ± 0.112	0.084
C10:1	0.412 ± 0.088	0.362 ± 0.090	5.588×10^{5}*	0.364 ± 0.093	7.308×10^{5}*	0.359 ± 0.091	1.056×10^{4}*
C10:2	0.064 ± 0.022	0.067 ± 0.025	0.413	0.070 ± 0.025	0.143	0.073 ± 0.028	0.055
C12	0.094 ± 0.029	0.082 ± 0.035	0.004*	0.083 ± 0.037	0.016*	0.087 ± 0.045	0.361
C12-DC	0.047 ± 0.007	0.047 ± 0.008	0.530	0.047 ± 0.008	0.515	0.047 ± 0.008	0.589
C12:1	0.311 ± 0.097	0.290 ± 0.097	0.143	0.288 ± 0.099	0.137	0.288 ± 0.104	0.229
C14	0.036 ± 0.011	0.034 ± 0.012	0.484	0.035 ± 0.013	0.750	0.037 ± 0.016	0.589
C14:1	0.071 ± 0.019	0.059 ± 0.018	5.588×10^{5}*	0.059 ± 0.018	7.308×10^{5}*	0.061 ± 0.021	0.003*
C14:1-OH	0.015 ± 0.005	0.014 ± 0.005	0.030*	0.014 ± 0.005	0.107	0.015 ± 0.006	0.598
C14:2	0.041 ± 0.024	0.036 ± 0.025	0.189	0.038 ± 0.026	0.403	0.042 ± 0.031	0.946
C14:2-OH	0.009 ± 0.004	0.008 ± 0.004	0.762	0.009 ± 0.004	0.899	0.009 ± 0.005	0.351
C16	0.099 ± 0.037	0.096 ± 0.043	0.686	0.100 ± 0.045	0.945	0.106 ± 0.051	0.438
C16-OH	0.006 ± 0.002	0.007 ± 0.002	0.638	0.007 ± 0.002	0.568	0.007 ± 0.002	0.275
C16:1	0.024 ± 0.011	0.022 ± 0.010	0.131	0.022 ± 0.010	0.386	0.024 ± 0.012	0.938
C16:1-OH	0.012 ± 0.003	0.012 ± 0.004	0.632	0.013 ± 0.004	0.463	0.013 ± 0.005	0.164
C16:2	0.020 ± 0.012	0.019 ± 0.012	0.479	0.019 ± 0.013	0.647	0.021 ± 0.015	0.764

C16:2-OH	0.015 ± 0.005	0.015 ± 0.006	0.762	0.015 ± 0.007	0.522	0.016 ± 0.008	0.229
C18	0.038 ± 0.011	0.035 ± 0.012	0.036*	0.036 ± 0.012	0.158	0.037 ± 0.013	0.596
C18:1	0.094 ± 0.050	0.091 ± 0.047	0.686	0.094 ± 0.048	0.992	0.100 ± 0.054	0.576
C18:1-OH	0.008 ± 0.002	0.008 ± 0.002	0.206	0.008 ± 0.003	0.425	0.008 ± 0.003	0.957
C18:2	0.059 ± 0.032	0.060 ± 0.036	0.813	0.062 ± 0.0372	0.543	0.065 ± 0.042	0.413
C2	6.099 ± 3.046	6.008 ± 2.994	0.847	6.215 ± 3.067	0.838	6.597 ± 3.589	0.445
C3	0.296 ± 0.093	0.346 ± 0.153	0.002*	0.354 ± 0.163	0.002*	0.365 ± 0.176	0.011*
C3-DC (C4-OH)	0.050 ± 0.022	0.052 ± 0.022	0.724	0.053 ± 0.023	0.547	0.051 ± 0.020	0.905
C3-OH	0.016 ± 0.010	0.020 ± 0.017	0.015*	0.021 ± 0.019	0.006*	0.023 ± 0.020	0.013*
C3:1	0.006 ± 0.002	0.006 ± 0.002	0.201	0.006 ± 0.002	0.193	0.006 ± 0.002	0.110
C4	0.169 ± 0.063	0.225 ± 0.094	5.588 × 10^{5}*	0.232 ± 0.099	7.308 × 10^{5}*	0.249 ± 0.105	1.056 × 10^{4}*
C4:1	0.027 ± 0.017	0.023 ± 0.014	0.078	0.022 ± 0.013	0.041*	0.021 ± 0.011	0.019*
C6 (C4:1-DC)	0.090 ± 0.030	0.090 ± 0.035	0.917	0.094 ± 0.037	0.483	0.101 ± 0.043	0.085
C5	0.105 ± 0.037	0.133 ± 0.081	5.588 × 10^{5}*	0.138 ± 0.086	7.308 × 10^{5}*	0.146 ± 0.106	0.009*
C5-M-DC	0.024 ± 0.005	0.027 ± 0.009	5.588 × 10^{5}*	0.027 ± 0.009	7.308 × 10^{5}*	0.028 ± 0.007	1.056 × 10^{4}*
C5-OH (C3-DC-M)	0.032 ± 0.008	0.035 ± 0.010	0.015*	0.036 ± 0.010	0.005*	0.036 ± 0.010	0.020*
C5:1	0.020 ± 0.005	0.019 ± 0.005	0.479	0.019 ± 0.005	0.451	0.018 ± 0.004	0.087
C5:1-DC	0.197 ± 0.220	0.109 ± 0.172	0.001*	0.091 ± 0.153	7.308 × 10^{5}*	0.060 ± 0.109	1.056 × 10^{4}*
C5-DC (C6-OH)	0.027 ± 0.010	0.031 ± 0.013	0.015*	0.032 ± 0.013	0.005*	0.032 ± 0.012	0.020*
C6:1	0.015 ± 0.004	0.016 ± 0.004	0.762	0.016 ± 0.004	0.451	0.016 ± 0.004	0.224
C7-DC	0.034 ± 0.014	0.029 ± 0.015	0.017*	0.030 ± 0.016	0.058	0.031 ± 0.018	0.331
C8	0.216 ± 0.105	0.179 ± 0.074	0.003*	0.182 ± 0.077	0.011*	0.187 ± 0.084	0.073
C9	0.025 ± 0.007	0.023 ± 0.009	0.055	0.023 ± 0.010	0.235	0.024 ± 0.010	0.513

**Amino acids and biogenic amines** (28)							
Alanine	305.756 ± 84.115	333.445 ± 108.850	0.037*	337.944 ± 114.548	0.027*	348.279 ± 124.768	0.032*
Arginine	85.039 ± 23.463	83.741 ± 29.820	0.762	84.162 ± 30.854	0.870	84.157 ± 32.328	0.906
Asparagine	38.112 ± 9.699	41.204 ± 12.467	0.043*	41.430 ± 12.554	0.044*	42.573 ± 13.789	0.045*
Aspartate	2.624 ± 2.198	4.038 ± 3.291	5.588 × 10^{5}*	4.290 ± 3.505	7.308 × 10^{5}*	4.794 ± 3.844	1.056 × 10^{4}*
Citrulline	23.919 ± 7.485	28.206 ± 13.782	0.003*	29.003 ± 14.350	0.002*	31.358 ± 16.362	0.002*
Glutamine	555.236 ± 97.640	557.110 ± 126.378	0.917	566.965 ± 129.689	0.547	573.038 ± 131.106	0.460
Glutamate	45.699 ± 19.049	59.787 ± 30.219	5.588 × 10^{5}*	61.350 ± 30.872	7.308 × 10^{5}*	61.109 ± 31.228	0.001*
Glycine	188.880 ± 61.530	194.551 ± 80.650	0.613	196.832 ± 84.891	0.522	197.899 ± 50.769	0.434
Histidine	70.724 ± 11.750	69.583 ± 15.922	0.599	70.362 ± 16.301	0.895	71.215 ± 16.872	0.905
Isoleucine	70.084 ± 17.091	76.435 ± 20.577	0.013*	77.666 ± 21.045	0.006*	78.524 ± 22.076	0.020*
Leucine	117.359 ± 30.687	120.911 ± 35.855	0.519	121.714 ± 37.253	0.451	120.077 ± 39.272	0.719
Lycine	206.701 ± 43.463	202.602 ± 50.145	0.583	203.000 ± 50.777	0.655	200.418 ± 45.726	0.488
Methionine	24.129 ± 4.861	22.812 ± 7.580	0.138	22.950 ± 7.788	0.246	23.307 ± 8.266	0.589
Ornithine	70.237 ± 30.908	82.678 ± 44.081	0.015*	83.742 ± 43.245	0.012*	86.626 ± 46.846	0.028*
Phenylalanine	62.966 ± 9.954	67.309 ± 15.943	0.014*	67.388 ± 16.209	0.022*	67.872 ± 17.536	0.073
Proline	155.384 ± 46.116	194.971 ± 76.197	5.588 × 10^{5}*	198.199± 77.406	7.308 × 10^{5}*	203.175 ± 79.634	1.056 × 10^{4}*
Serine	98.112 ± 23.126	97.173 ± 31.151	0.816	98.028 ± 32.155	0.992	100.671 ± 33.572	0.682
Theronine	105.313 ± 28.333	108.218 ± 34.033	0.556	109.315 ± 34.807	0.452	111.167 ± 33.895	0.338
Tryptophan	50.754 ± 10.146	46.753 ± 12.259	0.008*	46.718 ± 12.433	0.014*	47.248 ± 12.967	0.103
Tyrosine	58.550 ± 12.494	58.887 ± 17.428	0.888	59.320 ± 18.256	0.778	60.361 ± 19.951	0.617
Valine	201.213 ± 47.019	209.642 ± 55.678	0.279	213.261 ± 57.213	0.141	217.038 ± 61.916	0.125

Asymmetric demethylarginine	0.357 ± 0.100	0.409 ± 0.132	0.001*	0.421 ± 0.137	7.308×10^{5}*	0.432 ± 0.142	1.056×10^{4}*
alpha Aminoadipic acid	1.088 ± 0.526	1.143 ± 0.523	0.526	1.134 ± 0.510	0.612	1.113 ± 0.530	0.847
Kynurenine	1.735 ± 0.450	2.021 ± 0.655	5.588×10^{5}*	2.059 ± 0.668	7.308×10^{5}*	2.125 ± 0.704	1.056×10^{4}*
Sarcosine	2.016 ± 1.721	2.311 ± 1.738	0.262	2.453 ± 1.777	0.107	2.821 ± 1.922	0.012*
trans-4-Hydroxyproline	14.993 ± 8.139	14.409 ± 8.340	0.660	14.563 ± 8.473	0.778	15.082 ± 9.119	0.962
Taurine	59.401 ± 31.049	65.041 ± 32.783	0.241	66.817 ± 33.839	0.143	70.760 ± 38.611	0.080
Symmetric dimethylarginine	0.461 ± 0.116	0.571 ± 0.260	5.588×10^{5}*	0.590 ± 0.277	7.308×10^{5}*	0.607 ± 0.297	1.056×10^{4}*
**Glycerophospholipids** (81)							
lysoPC a C16:0	76.291 ± 27.730	79.677 ± 31.270	0.479	82.635 ± 30.568	0.161	88.630 ± 31.211	0.019*
lysoPC a C16:1	1.787 ± 0.858	1.935 ± 0.977	0.295	2.009 ± 1.001	0.130	2.203 ± 1.135	0.025*
lysoPC a C17:0	1.269 ± 0.523	1.265 ± 0.584	0.961	1.319 ± 0.594	0.612	1.424 ± 0.642	0.164
lysoPC a C18:0	24.897 ± 8.515	24.524 ± 10.252	0.799	25.540 ± 10.414	0.703	27.357 ± 11.350	0.217
lysoPC a C18:1	12.982 ± 5.416	13.644 ± 5.764	0.471	14.155 ± 5.654	0.170	15.468 ± 5.655	0.012*
lysoPC a C18:2	26.330 ± 10.447	25.849 ± 11.860	0.786	26.838 ± 11.889	0.805	28.074 ± 11.963	0.434
lysoPC a C20:3	1.385 ± 0.645	1.580 ± 0.779	0.048*	1.649 ± 0.780	0.011*	1.794 ± 0.855	0.003*
lysoPC a C20:4	4.319 ± 1.993	5.122 ± 2.399	0.006*	5.337 ± 2.368	0.001*	5.697 ± 2.451	1.056×10^{4}*
lysoPC a C24:0	0.145 ± 0.038	0.142 ± 0.040	0.583	0.146 ± 0.040	0.939	0.154 ± 0.043	0.251
lysoPC a C26:0	0.108 ± 0.030	0.113 ± 0.032	0.322	0.115 ± 0.033	0.143	0.121 ± 0.032	0.020*
lysoPC a C26:1	0.054 ± 0.017	0.054 ± 0.019	0.917	0.056 ± 0.020	0.647	0.058 ± 0.020	0.251
lysoPC a C28:1	0.158 ± 0.065	0.147 ± 0.053	0.241	0.151 ± 0.055	0.483	0.160 ± 0.052	0.914
PC aa C24:0	0.035 ± 0.018	0.052 ± 0.166	0.296	0.055 ± 0.183	0.345	0.069 ± 0.244	0.363
PC aa C28:1	1.163 ± 0.520	1.019 ± 0.411	0.031*	1.058 ± 0.416	0.154	1.119 ± 0.432	0.669
PC aa C30:0	1.528 ± 0.695	1.435 ± 0.733	0.414	1.508 ± 0.766	0.891	1.626 ± 0.818	0.530
PC aa C32:0	7.825 ± 1.987	7.614 ± 2.114	0.530	7.839 ± 2.178	0.981	8.087 ± 2.384	0.564

PC aa C32:1	5.054 ± 3.671	5.238 ± 4.116	0.762	5.466 ± 4.417	0.547	5.784 ± 4.221	0.351
PC aa C32:3	0.192 ± 0.051	0.170 ± 0.048	0.001*	0.175 ± 0.050	0.020*	0.177 ± 0.052	0.100
PC aa C34:1	99.384 ± 21.357	102.072 ± 24.836	0.479	104.073 ± 25.627	0.196	108.366 ± 28.338	0.052
PC aa C34:2	132.055 ± 42.922	132.206 ± 44.514	0.977	135.064 ± 45.928	0.703	140.487 ± 49.121	0.351
PC aa C34:3	6.317 ± 2.578	5.956 ± 2.719	0.389	6.145 ± 2.864	0.725	6.111 ± 2.377	0.685
PC aa C34:4	0.625 ± 0.228	0.562 ± 0.256	0.071	0.583 ± 0.263	0.297	0.599 ± 0.269	0.613
PC aa C36:0	2.775 ± 1.219	2.253 ± 0.954	5.588×10^{5}*	2.249 ± 0.967	0.001*	2.249 ± 0.965	0.005*
PC aa C36:1	27.774 ± 10.128	26.191 ± 11.522	0.353	27.069 ± 12.141	0.725	28.209 ± 11.507	0.872
PC aa C36:2	109.595 ± 29.310	106.416 ± 27.098	0.479	107.933 ± 27.366	0.745	110.984 ± 30.370	0.847
PC aa C36:3	58.759 ± 15.554	58.504 ± 18.064	0.918	60.135 ± 18.513	0.647	62.238 ± 18.269	0.283
PC aa C36:4	92.354 ± 18.510	97.761 ± 23.988	0.068	100.182 ± 24.102	0.011*	101.266 ± 26.364	0.034*
PC aa C36:5	12.719 ± 8.789	10.791 ± 7.910	0.101	11.444 ± 8.368	0.377	12.631 ± 10.091	0.963
PC aa C36:6	0.390 ± 0.184	0.313 ± 0.176	0.001*	0.326 ± 0.184	0.016*	0.350 ± 0.216	0.304
PC aa C38:0	2.514 ± 0.759	2.151 ± 0.667	5.588×10^{5}*	2.185 ± 0.673	0.001*	2.250 ± 0.725	0.047*
PC aa C38:3	24.875 ± 9.195	26.188 ± 9.297	0.363	27.046 ± 9.683	0.143	28.114 ± 9.611	0.052
PC aa C38:4	57.115 ± 16.725	62.106 ± 22.091	0.065	64.051 ± 22.949	0.016*	65.454 ± 25.619	0.039*
PC aa C38:5	24.332 ± 7.606	23.732 ± 8.454	0.641	24.573 ± 8.702	0.885	25.968 ± 9.600	0.351
PC aa C38:6	58.764 ± 17.351	54.764 ± 16.471	0.094	55.884 ± 16.981	0.303	56.827 ± 18.151	0.596
PC aa C40:2	0.354 ± 0.258	0.285 ± 0.176	0.029*	0.295 ± 0.190	0.092	0.324 ± 0.241	0.575
PC aa C40:3	0.500 ± 0.295	0.446 ± 0.207	0.148	0.462 ± 0.221	0.386	0.507 ± 0.279	0.932
PC aa C40:4	2.024 ± 0.639	2.058 ± 0.800	0.762	2.110 ± 0.838	0.483	2.170 ± 0.865	0.351
PC aa C40:5	5.349 ± 1.725	5.312 ± 2.001	0.906	5.483 ± 2.076	0.693	5.720 ± 2.149	0.351
PC aa C40:6	22.433 ± 7.213	21.735 ± 8.006	0.573	22.338 ± 8.400	0.951	23.045 ± 9.561	0.739
PC aa C42:0	0.452 ± 0.140	0.428 ± 0.148	0.284	0.434 ± 0.151	0.475	0.452 ± 0.164	0.994
PC aa C42:1	0.266 ± 0.076	0.246 ± 0.082	0.082	0.250 ± 0.083	0.193	0.257 ± 0.087	0.596

PC aa C42:2	0.278 ± 0.105	0.225 ± 0.078	5.588 × 10^{5}*	0.230 ± 0.082	7.308 × 10^{5}*	0.238 ± 0.090	0.026*
PC aa C42:4	0.170 ± 0.039	0.156 ± 0.039	0.006*	0.158 ± 0.040	0.038*	0.162 ± 0.043	0.317
PC aa C42:5	0.276 ± 0.192	0.230 ± 0.097	0.039*	0.236 ± 0.101	0.098	0.252 ± 0.119	0.434
PC aa C42:6	0.332 ± 0.228	0.283 ± 0.143	0.078	0.291 ± 0.150	0.167	0.315 ± 0.187	0.685
PC ae C30:0	0.153 ± 0.041	0.132 ± 0.043	5.588 × 10^{5}*	0.136 ± 0.045	0.006*	0.142 ± 0.050	0.224
PC ae C30:2	0.046 ± 0.012	0.041 ± 0.010	5.588 × 10^{5}*	0.042 ± 0.010	0.003*	0.043 ± 0.010	0.050
PC ae C32:1	1.341 ± 0.372	1.150 ± 0.354	5.588 × 10^{5}*	1.161 ± 0.365	7.308 × 10^{5}*	1.199 ± 0.382	0.033*
PC ae C32:2	0.368 ± 0.098	0.302 ± 0.090	5.588 × 10^{5}*	0.306 ± 0.093	7.308 × 10^{5}*	0.310 ± 0.099	1.056 × 10^{4}*
PC ae C34:0	0.644 ± 0.164	0.578 ± 0.176	0.004*	0.592 ± 0.176	0.038*	0.616 ± 0.196	0.423
PC ae C34:1	3.798 ± 0.927	3.698 ± 1.084	0.540	3.777 ± 1.105	0.915	3.982 ± 1.138	0.377
PC ae C34:2	6.165 ± 1.558	5.115 ± 1.682	5.588 × 10^{5}*	5.216 ± 1.721	7.308 × 10^{5}*	5.294 ± 1.798	0.003*
PC ae C34:3	4.553 ± 1.334	3.539 ± 1.235	5.588 × 10^{5}*	3.612 ± 1.250	7.308 × 10^{5}*	3.634 ± 1.226	1.056 × 10^{4}*
PC ae C36:0	0.536 ± 0.157	0.470 ± 0.125	0.001*	0.479 ± 0.128	0.006*	0.486 ± 0.132	0.060
PC ae C36:1	7.635 ± 2.910	7.089 ± 3.096	0.227	7.269 ± 3.120	0.475	7.542 ± 3.329	0.906
PC ae C36:2	7.429 ± 1.663	6.534 ± 1.866	5.588 × 10^{5}*	6.668 ± 1.910	0.003*	6.823 ± 2.088	0.082
PC ae C36:3	3.785 ± 0.939	3.235 ± 0.980	5.588 × 10^{5}*	3.311 ± 1.007	7.308 × 10^{5}*	3.386 ± 1.027	0.021*
PC ae C36:4	9.274 ± 2.807	8.197 ± 2.472	0.002*	8.352 ± 2.482	0.016*	8.395 ± 2.558	0.073
PC ae C36:5	7.123 ± 2.443	6.034 ± 1.864	5.588 ×	6.166 ± 1.894	0.002*	6.293 ± 1.939	0.030*

			10^{5}*				
PC ae C38:0	0.994 ± 0.355	0.831 ± 0.323	5.588×10^{5}*	0.856 ± 0.335	0.006*	0.900 ± 0.392	0.172
PC ae C38:2	1.020 ± 0.457	0.902 ± 0.568	0.101	0.931 ± 0.599	0.303	0.989 ± 0.680	0.831
PC ae C38:3	2.821 ± 0.733	2.749 ± 0.854	0.580	2.819 ± 0.880	0.992	2.904 ± 0.971	0.648
PC ae C38:4	5.602 ± 1.452	5.548 ± 1.519	0.813	5.661 ± 1.564	0.838	5.816 ± 1.779	0.538
PC ae C38:5	8.332 ± 2.259	7.766 ± 1.991	0.056	7.909 ± 2.020	0.200	8.147 ± 2.141	0.685
PC ae C38:6	4.390 ± 1.480	3.600 ± 1.075	5.588×10^{5}*	3.668 ± 1.113	7.308×10^{5}*	3.736 ± 1.156	0.004*
PC ae C40:1	0.852 ± 0.269	0.751 ± 0.324	0.013*	0.775 ± 0.332	0.096	0.811 ± 0.374	0.564
PC ae C40:2	1.124 ± 0.356	1.043 ± 0.356	0.109	1.074 ± 0.359	0.412	1.117 ± 0.399	0.946
PC ae C40:3	0.898 ± 0.256	0.857 ± 0.278	0.318	0.873 ± 0.284	0.596	0.898 ± 0.315	0.994
PC ae C40:4	1.249 ± 0.282	1.191 ± 0.303	0.187	1.210 ± 0.313	0.451	1.245 ± 0.352	0.957
PC ae C40:5	2.259 ± 0.609	2.097 ± 0.604	0.058	2.129 ± 0.607	0.167	2.197 ± 0.660	0.635
PC ae C40:6	2.894 ± 0.831	2.634 ± 0.744	0.015*	2.687 ± 0.747	0.089	2.788 ± 0.810	0.538
PC ae C42:0	0.513± 0.129	0.497 ± 0.158	0.507	0.502 ± 0.169	0.703	0.517 ± 0.203	0.914
PC ae C42:1	0.260 ± 0.074	0.249 ± 0.082	0.398	0.255 ± 0.086	0.749	0.263 ± 0.094	0.883
PC ae C42:2	0.357 ± 0.095	0.326 ± 0.120	0.031*	0.335 ± 0.120	0.171	0.350 ± 0.134	0.779
PC ae C42:3	0.590 ± 0.163	0.526 ± 0.193	0.009*	0.537 ± 0.195	0.045*	0.557 ± 0.216	0.393
PC ae C42:4	0.528 ± 0.163	0.495 ± 0.179	0.201	0.505 ± 0.186	0.451	0.530 ± 0.205	0.957
PC ae C42:5	1.187 ± 0.271	1.171 ± 0.327	0.735	1.180 ± 0.332	0.898	1.224 ± 0.361	0.596
PC ae C44:3	0.085 ± 0.023	0.078 ± 0.026	0.045*	0.079 ± 0.027	0.139	0.082 ± 0.029	0.638
PC ae C44:4	0.213 ± 0.056	0.210 ± 0.069	0.741	0.213 ± 0.072	0.981	0.222 ± 0.078	0.538
PC ae C44:5	0.804 ± 0.231	0.823 ± 0.284	0.638	0.834 ± 0.291	0.483	0.871 ± 0.313	0.217
PC ae C44:6	0.845 ± 0.261	0.851 ± 0.261	0.885	0.868 ± 0.269	0.613	0.903 ± 0.284	0.271
**Sphingolipids** (14)							
SM (OH) C14:1	4.145 ± 1.446	3.817 ± 1.098	0.078	3.950 ± 1.093	0.368	4.200 ± 1.119	0.857

SM (OH) C16:1	2.965 ± 1.009	2.903 ± 0.793	0.683	2.991 ± 0.784	0.885	3.119 ± 0.782	0.368
SM (OH) C22:1	21.141 ± 11.388	19.858 ± 9.184	0.451	20.398 ± 9.187	0.693	20.747 ± 8.753	0.872
SM (OH) C22:2	18.226 ± 11.455	18.157 ± 9.529	0.961	18.590 ± 9.512	0.860	19.123 ± 9.269	0.669
SM (OH) C24:1	1.364 ± 0.484	1.294 ± 0.354	0.295	1.342 ± 0.342	0.778	1.394 ± 0.370	0.726
SM C16:0	120.573 ± 37.978	122.613 ± 33.164	0.724	126.322 ± 32.656	0.319	134.352 ± 33.086	0.032*
SM C16:1	17.042 ± 5.482	17.232 ± 4.839	0.813	17.815 ± 4.688	0.366	18.704 ± 4.907	0.080
SM C18:0	24.180 ± 9.748	25.760 ± 9.943	0.295	26.392 ± 10.181	0.153	27.106 ± 10.093	0.100
SM C18:1	11.940 ± 4.440	12.954 ± 4.228	0.098	13.308 ± 4.218	0.031*	13.599 ± 4.311	0.033*
SM C20:2	0.440 ± 0.168	0.465 ± 0.160	0.303	0.477 ± 0.161	0.148	0.484 ± 0.172	0.164
SM C24:0	29.005 ± 11.095	28.008 ± 9.684	0.542	28.845 ± 9.625	0.939	30.072 ± 9.639	0.626
SM C24:1	96.354 ± 52.961	104.571 ± 51.648	0.301	106.859 ± 52.132	0.196	112.930 ± 52.966	0.082
SM C26:0	0.241 ± 0.091	0.231 ± 0.073	0.451	0.238 ± 0.073	0.838	0.246 ± 0.072	0.773
SM C26:1	0.527 ± 0.233	0.539 ± 0.209	0.735	0.559 ± 0.217	0.392	0.604 ± 0.254	0.073

* indicates P value < 0.05.
