# Supplementary figures and images for: Identification of Metabolomics Biomarkers in Extracranial Carotid Artery Stenosis
Source: Cells. 2022 Sep 27;11(19):3022. doi: 10.3390/cells11193022 (PMC9563778; doi:10.3390/cells11193022)

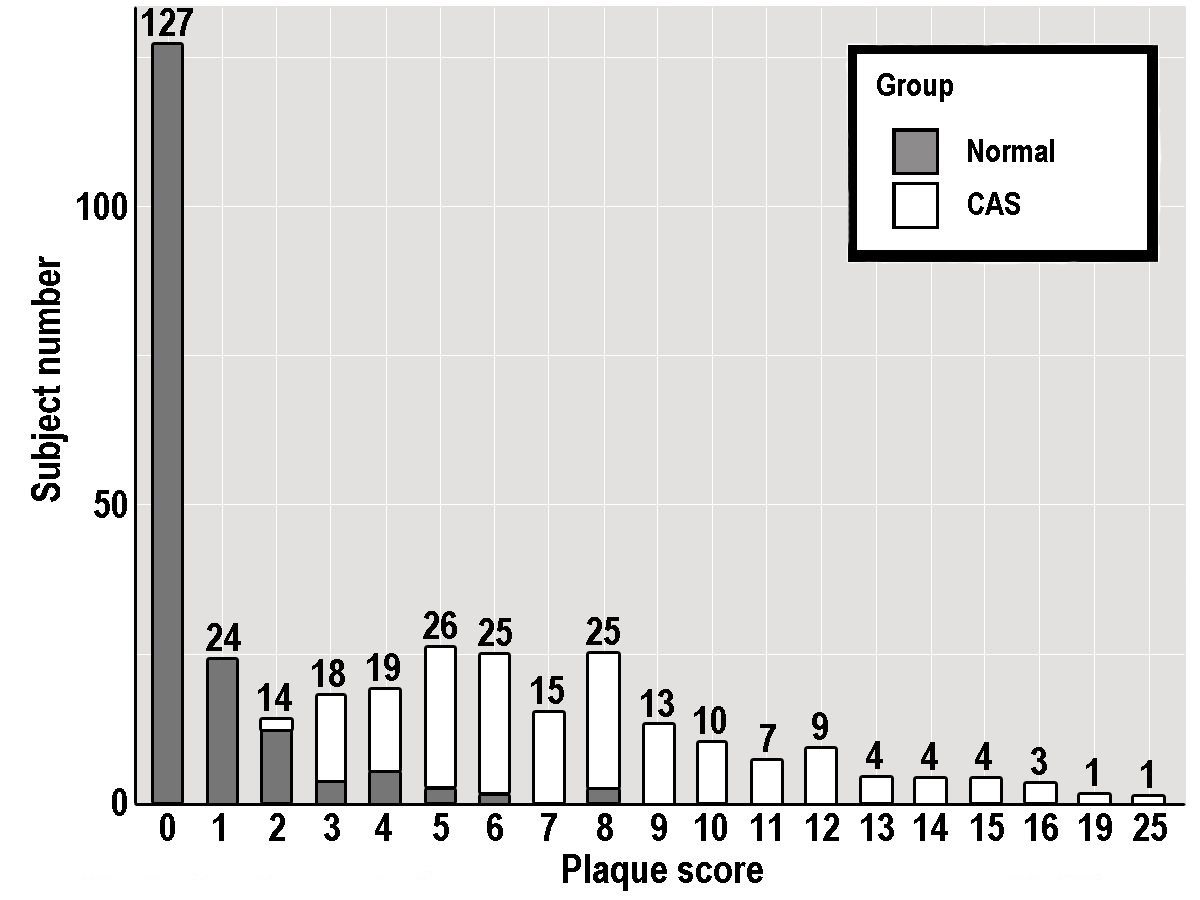

Supplement: Supplementary file 1 [file cells-11-03022-s001.zip › Supplementary Figure S1, plaque score_349_F.tif]

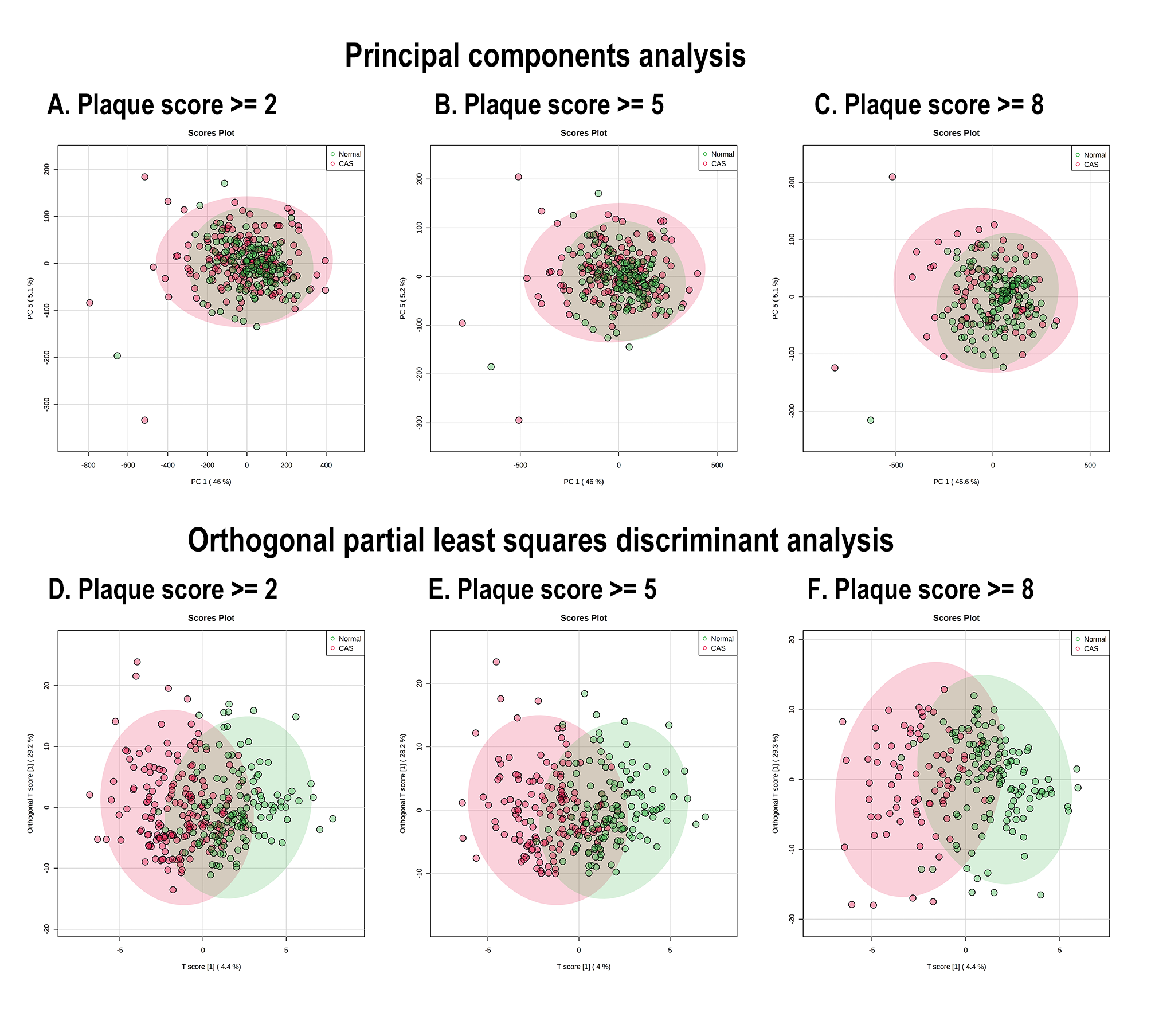

Supplement: Supplementary file 1 [file cells-11-03022-s001.zip › Supplementary Figure S2, PCA and PLSDA.tif]

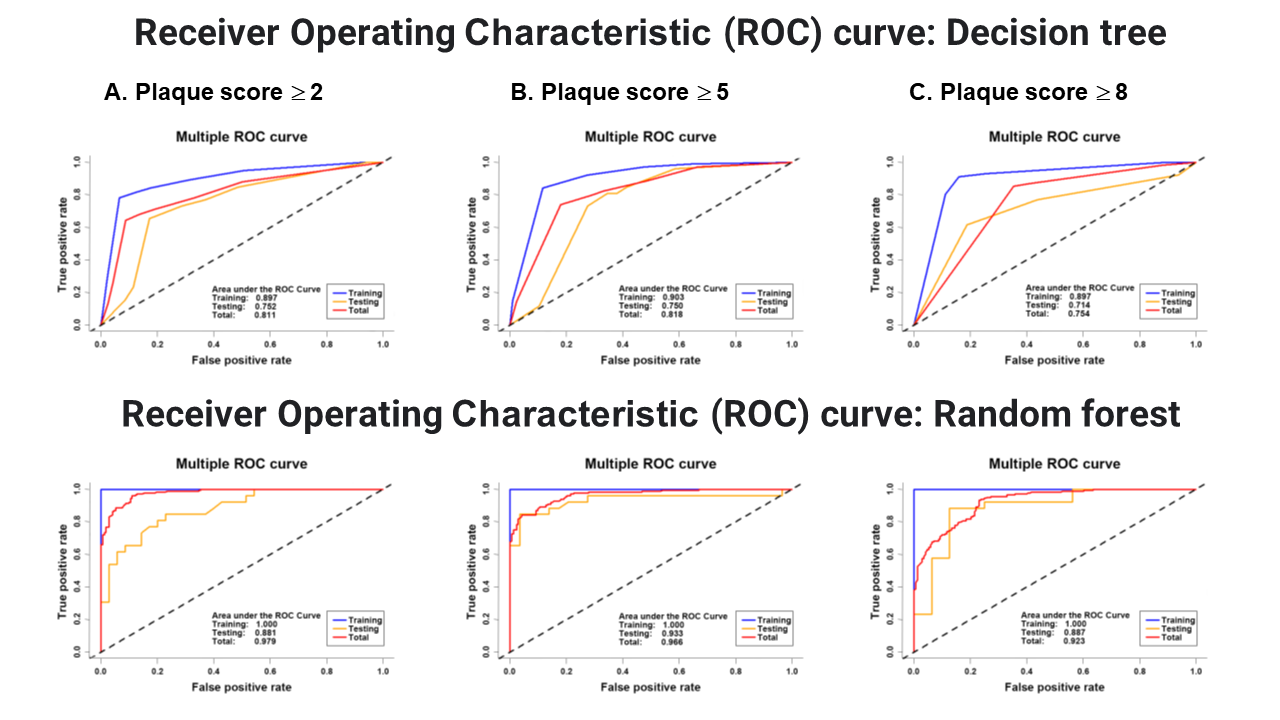

Supplement: Supplementary file 1 [file cells-11-03022-s001.zip › Supplementary Figure S3- ROC.TIF]
